# Early Environmental Origins of Neurodegenerative Disease in Later Life

**DOI:** 10.1289/ehp.7571

**Published:** 2005-05-26

**Authors:** Philip J. Landrigan, Babasaheb Sonawane, Robert N. Butler, Leonardo Trasande, Richard Callan, Daniel Droller

**Affiliations:** 1 Center for Children’s Health and the Environment, Department of Community and Preventive Medicine, Mount Sinai School of Medicine, New York, New York, USA; 2 National Center for Environmental Assessment, Office of Research and Development, U.S. Environmental Protection Agency, Washington, DC, USA; 3 International Longevity Center, New York, New York, USA

**Keywords:** Alzheimer disease, maneb, manganese, National Children’s Study, neurodegenerative disease, paraquat, Parkinson disease, pesticides

## Abstract

Parkinson disease (PD) and Alzheimer disease (AD), the two most common neurodegenerative disorders in American adults, are of purely genetic origin in a minority of cases and appear in most instances to arise through interactions among genetic and environmental factors. In this article we hypothesize that environmental exposures in early life may be of particular etiologic importance and review evidence for the early environmental origins of neurodegeneration. For PD the first recognized environmental cause, MPTP (1-methyl-4-phenyl-1,2,3,6-tetrahydropyridine), was identified in epidemiologic studies of drug abusers. Chemicals experimentally linked to PD include the insecticide rotenone and the herbicides paraquat and maneb; interaction has been observed between paraquat and maneb. In epidemiologic studies, manganese has been linked to parkinsonism. In dementia, lead is associated with increased risk in chronically exposed workers. Exposures of children in early life to lead, polychlorinated biphenyls, and methylmercury have been followed by persistent decrements in intelligence that may presage dementia. To discover new environmental causes of AD and PD, and to characterize relevant gene–environment interactions, we recommend that a large, prospective genetic and epidemiologic study be undertaken that will follow thousands of children from conception (or before) to old age. Additional approaches to etiologic discovery include establishing incidence registries for AD and PD, conducting targeted investigations in high-risk populations, and improving testing of the potential neurologic toxicity of chemicals.

Parkinson disease (PD) and Alzheimer disease (AD) are the two most common neurodegenerative diseases of the older American population. PD affects more than 500,000 Americans (National Institute of Neurological Disorders and Stroke 2004; Siderowf and Stern 2003). About 50,000 new cases are reported each year, and in recent years the annual number of deaths from PD has increased steadily (Lilienfeld et al. 1990). Internationally, the incidence rate for PD approximates 17 per 100,000 per year, although this is probably an underestimate (Twelves et al. 2003). AD has been diagnosed in an estimated 2.3 million persons in the United States, and there are approximately 360,000 newly diagnosed cases each year (Brookmeyer et al. 1998). It is estimated that by 2050, as the U.S. population continues to age, as many as 14 million Americans may have AD (Lewin Group 2001).

Causation of both PD and AD is complex. In a minority of cases, particularly in early onset AD and PD, etiology appears to be primarily genetic (Tanner et al. 1999). But in most cases, causation appears to involve interactions among multiple genetic and environmental factors (Foster 2002; Kennedy et al. 2003). We hypothesize that exposure of the developing brain to still undefined toxic environmental agents during windows of vulnerability in early life—*in utero* and in early postnatal life—may be an important contributor to causation.

Here we provide an overview of the emerging body of evidence on the environmental origins of neurodegenerative disease. We focus especially on environmental exposures that occur early in life during windows of developmental vulnerability. We offer recommendations for future research. This report and its recommendations are based on the conference “Early Environmental Origins of Neurodegenerative Disease in Later Life: Research and Risk Assessment” sponsored by the Mount Sinai Center for Children’s Health and the Environment. The conference was held in New York City on 16 May 2003.

## The Pathology of PD and AD

PD presents clinically as a disorder of motor function characterized by tremor, slow and decreased movement (bradykinesia), muscular rigidity, poor balance, and problems in gait (Parkinson’s Disease Foundation 2004). Pathologically, PD patients show loss of dopaminergic neurons in the substantia nigra (SN) pars compacta and frequently have Lewy bodies, eosinophilic intracellular inclusions composed of amyloid-like fibers and α-synuclein (Dawson and Dawson 2003).

AD is characterized by a deterioration of cortical neurons, resulting in dementia. The two typical histopathologic features are *a*) plaques, which are clumps of insoluble β-amyloid protein fragments that accumulate extracellularly, and *b*) intracellular neurofibrillary tangles composed of altered tau protein (Alzheimer’s Association 2003).

## Costs of Neurogenerative Disease

A 1997 economic study prepared for the Parkinson’s Disease Foundation estimated the annual cost of treatment per patient to be approximately $24,000 (John C. Robbins Associates 1997). The estimated total annual costs of treating PD in the United States range from $12 to 25 billion. These costs are spread across families, benefit providers, social security, Medicare, and Medicaid. In addition to the financial costs, there are the human costs of pain and suffering, sadness and despair, and reduction in overall quality of life.

Combined Medicare and Medicaid spending on AD amounted to more than $50 billion in 2000 and is anticipated to increase to nearly $83 billion by 2010 (Lewin Group 2001). Preliminary statistics from 2001—the most recent year for which these data are available—from the Centers for Disease Control and Prevention (CDC) list AD as the eighth leading cause of death in the United States, responsible for 62,000 deaths annually (CDC 2003a).

PD and AD may co-occur and may share some etiologic or predisposing factors. Elderly patients who develop rapidly progressive PD may be at up to 8 times increased risk of developing AD (Wilson et al. 2003). Although the risk of developing AD and PD increases with age, neither of these diseases nor the symptoms of dementia are part of normal aging. In the absence of disease, the human brain can function well into the tenth decade [National Institute on Aging (NIA) 2000].

## The Barker Hypothesis

Through detailed reconstructions of neonatal and medical histories of birth cohorts in the United Kingdom, David Barker of the University of Southampton proposed what is now termed “the Barker hypothesis” (Osmond and Barker 2000), the concept that parameters of fetal, infant, and childhood growth may be predictors of disease in later life. Barker found that infants with low birth weight, small head circumference, and low ponderal index at birth are at increased risk of developing coronary heart disease, hypertension, stroke, insulin resistance, and diabetes as adults. He found also that reduced fetal growth and impaired development during infancy were associated with increased mortality from cardiovascular disease (CVD) in both men and women, independent of social class and other confounders such as smoking, alcohol consumption, and obesity (Barker et al. 1993; Osmond et al. 1993). This association is strong and graded, is observed in various populations, and is specific to CVD. In Barker’s studies, low birth weight followed by obesity in later life led to a particularly high risk of CVD and insulin resistance. Further analysis indicated that hypertension may begin *in utero* and become magnified with age (Law et al. 1993).

Barker hypothesized that fetal undernutrition during critical periods of vulnerability in early development leads to persistent changes in hormone levels and in altered tissue sensitivity to these hormones, permanently altering the metabolism and body structure (Hinchliffe et al. 1992; Lumbers et al. 2001).

## The Expanded Barker Hypothesis

At the 2003 Mount Sinai Conference on Early Environmental Origins of Neurological Degeneration, we explored the plausibility of extending the Barker hypothesis to encompass brain development and to explore the impacts of toxic chemicals on brain development.

**Figure 1:**
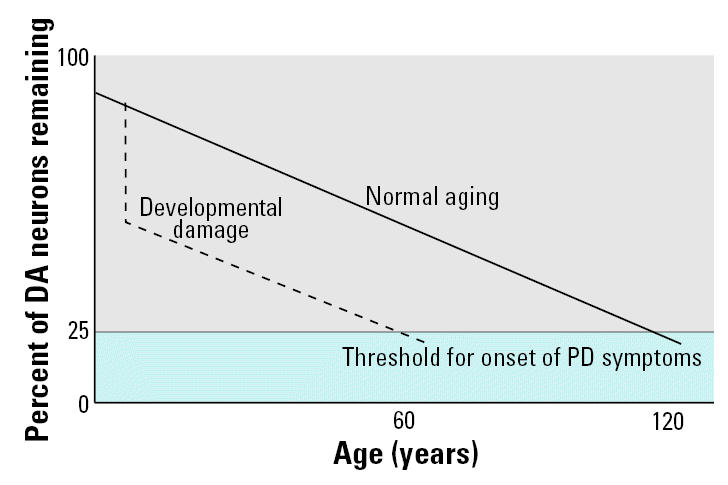
Long-term consequences of early loss of critical neurons after developmental damage. DA, dopaminergic. The impact of early developmental damage is not immediately evident but produces disease years or decades later as the number of neurons decreases with advancing age.

Conferees generally supported the hypothesis that early exposures to environmental toxicants could later affect the brain and that such associations are biologically plausible (De la Fuente-Fernandez and Calne 2002). This consensus was based on experimental studies of associations between early-life exposures to pesticides and PD (Thiruchelvam et al. 2000a, 2000b), as well as on epidemiologic studies of the toxic and apparently irreversible effects on the developing brain of *in utero* exposures to lead, methylmercury, and polychlorinated biphenyls (Grandjean et al. 1997; Jacobson et al. 1990; Needleman et al. 1990). A mechanistic hypothesis proposed (Langston et al. 1999) that early exposures to neurotoxic chemicals reduce the number of neurons in critical areas of the brain such as the SN to levels below those needed to sustain function in the face of the neuronal attrition associated with advancing age (Figure 1).

## Evidence for the Environmental Origins of Parkinson Disease

### Twin studies.

A large-scale study of twins designed to assess genetic versus environmental factors in the etiology of PD found a high degree of concordance within twin pairs for early-onset PD (onset before age 50) but much less concordance for disease of late onset (Tanner et al. 1999). This finding suggests that early onset PD may be of genetic origin in most cases (although the etiologic role of a shared environment can never be completely excluded), whereas beyond 50 years of age environmental factors become increasingly important (Tanner et al. 1999).

### MPTP and PD.

Several clinical and epidemiologic studies have demonstrated that exposures to certain synthetic chemicals are associated with increased incidence of PD. The first of these studies was the description in 1982 of severe Parkinson-like symptoms among a group of drug users in northern California who had taken synthetic heroin contaminated with MPTP (1-methyl-4-phenyl-1,2,3,6-tetrahydropyridine; Langston et al. 1999). This episode strongly supported the concept that exogenous chemicals can cause or contribute to causation of PD (Priyadarshi et al. 2001). MPTP was subsequently shown to act selectively—specifically injuring dopaminergic neurons in the nigrostriatal system in humans as well as in experimental animals (Langston et al. 1999). Evidence also was found for ongoing dopaminergic nerve cell loss without Lewy body formation in these patients. This suggested a self-perpetuating process of neurodegeneration. Years later, consistent with that hypothesis, postmortem examination of persons who had been exposed to MPTP showed a marked microglial proliferation in the SN pars compacta (Orr et al. 2002). In some patients, MPTP-induced PD appeared almost immediately after exposure, whereas in others, onset became evident only months or years later, apparently reflecting progressive injury against a background of declining physiologic reserve.

### Paraquat and PD.

An etiologic link has been suggested between PD and the herbicide paraquat (1,1′-dimethyl-4,4′-bipyridinium; Brooks et al. 1999; McCormack et al. 2002). Paraquat is structurally similar to MPP^+^, the active metabolite of MPTP. Epidemiologic data suggest a positive dose–response relationship between lifetime cumulative exposure to paraquat and risk of PD (Liou et al. 1997). In experimental studies in which paraquat has been administered to animals, researchers have observed loss of SN dopaminergic neurons, depletion of dopamine in the SN, reduced ambulatory activity, and apoptotic cell death (Liu et al. 2003).

### Maneb and PD.

Exposure to the dithiocarbamate fungicide maneb has been reported to enhance uptake of MPTP and to amplify its neurotoxicity; both paraquat and maneb target brain dopamine. In animal studies, early-life exposure to a combination of paraquat and maneb produced destructive effects on the nigrostriatal dopaminergic system and abnormalities in motor response that were more severe than those produced by either agent alone. These effects were amplified by aging (McCormack et al. 2002; Thiruchelvam et al. 2000a, 2000b).

### Rotenone and PD.

The insecticide rotenone induces clinical and pathologic features in rats similar to those induced by PD, including selective degeneration of the nigrostriatal dopaminergic system and movement disorders (Liu et al. 2003; Sherer et al. 2003). Synergistic effects have been observed in animals administered a combination of rotenone and lipopolysacchide, a molecule that stimulates inflammation (Gao et al. 2003; Thiruchelvam et al. 2000b).

### Manganese and PD.

Although manganese is an essential trace element, chronic occupational exposure to high levels of this metal causes accumulation in the basal ganglia, resulting in manganism, a condition characterized by tremors, rigidity and psychosis (Mergler and Baldwin 1997). This condition has been reported in manganese miners. Concern exists that widespread introduction of the manganese-containing fuel additive MMT (methyl-cyclopentadienyl manganese tricarbonyl) to the U.S. gasoline supply may increase population exposure to manganese and thus increase risk of parkinsonism in sensitive populations (Needleman and Landrigan 1996).

### Other chemicals and PD.

Exposures to pesticides and other organic compounds are widespread in the American population (CDC 2003b). Levels of organochlorines have been found to be elevated in the brains of persons with PD (Fleming et al. 1994). A study of French elderly individuals found an association between past occupational exposure to pesticides, low cognitive performance, and increased risk of developing AD or PD (Baldi et al. 2003). Other reported links between environmental factors and PD include increased risks from drinking well water, rural living, farming, and exposure to agricultural chemicals (Liou et al. 1997; Priyadarshi et al. 2001).

Epidemiologic studies have shown inverse, apparently protective relationships between cigarette smoking, coffee consumption, and PD (Hernan et al. 2002).

### Inflammation and PD.

Inflammation of the brain in early life caused by exposure to infectious agents, toxicants, or environmental factors has been suggested as a possible cause or contributor to the later development of PD (Liu et al. 2003). The inflammatory process in such cases may involve activation of brain immune cells (microglia and astrocytes), which release inflammatory and neurotoxic factors that in turn produce neurodegeneration (Liu and Hong 2003). This concept first arose in the suggestion that infection with influenza virus in the pandemic of 1918 produced an increased risk of PD. More recently, infection with certain microorganisms such as the soil bacterium *Nocardia asteroides* has been proposed as a risk factor for PD (Kohbata and Beaman 1991). In animal experiments, exposure to bacterial endotoxin lipopolysaccharide *in utero* induced dopaminergic neurodegeneration (Gao et al. 2002; Liu et al. 2000, 2003).

### Isolated populations of high risk for PD.

PD incidence and mortality rates differ among ethnic groups and exhibit strong regional variation, thus providing additional evidence that environmental factors may be involved in causation (Ben-Shlomo 1997; Foster 2002).

For example, the Chamorros population of Guam and Rota in the western Pacific have an unusually high prevalence of motor neuron disease, a syndrome that includes amyotrophic lateral sclerosis (ALS), parkinsonism, and progressive dementia. It has been proposed that this syndrome of parkinsonian dementia is related to the consumption of flour made from cycad seeds (Spencer 2003) or to inhalation of pollen from cycad plants (Seawright et al. 1995). Consumption of cycad flour may have been especially common on Guam in the famine years before and during World War II. The declining incidence and increasing age at onset of ALS and parkinsonism–dementia complex among the Chamorros over the past 50 years together with the decreasing prevalence of ALS over the same time in high-incidence areas of Japan and Indonesia suggests the disappearance of an environmental factor unique to these population groups (Kurland and Mulder 1954; Plato et al. 2003).

## Evidence for the Environmental Origins of Dementia

### Lead and cognitive function.

Childhood exposure to lead, even at relatively low levels (Canfield et al. 2003), results in a decline of cognitive function that persists into adulthood and that manifests as a persistent lowering of IQ score plus alteration in behavior (Needleman et al. 1990). Each increase of 10 μg/dL in the lifetime average blood lead concentration was found to be associated with a 4.6-point decrease in IQ (Schwartz et al. 2000). There appears to be no minimum threshold level below which lead does not cause brain injury (Canfield et al. 2003). In addition, elevated lead levels in childhood have been associated with lower class standing in high school, lower vocabulary and grammatical-reasoning scores, poorer hand–eye coordination, and self-reports of minor delinquent activity (Needleman et al. 1990).

Occupational exposure to lead among adults is associated with poorer neurobehavioral test scores and with deficits in manual dexterity, executive ability, verbal intelligence, and verbal memory (Schwartz et al. 2000). Recent data suggest that cognitive function can decline progressively in older lead workers in relation to cumulative past occupational exposure to lead (Stewart et al. 1999). Susceptibility to the persistent effect of lead on the central nervous system may be enhanced in persons who have at least one apolipoprotein E-4 allele (Stewart et al. 2002).

## Recommendations

The conferees agreed on recommendations for future research into the environmental etiology of chronic neurodegenerative disease.

### Conduct long-term prospective epidemiologic and genetic studies of the impact of environmental factors on the development of neurodegeneration.

Most previous research on the causation of the neurodegenerative disorders has been either cross-sectional or retrospective in design and thus has been extremely limited in its ability to discern environmental etiologic factors that may have been encountered in early life. Most previous studies have had to reconstruct past exposures from imperfect memory, from incomplete records, or from biologic markers of uncertain half-life. The conferees offered the suggestion that a large prospective cohort study would provide a most powerful tool to explore possible early environmental causes of neurodegenerative disease. If such a study were to include genetic analyses, it would provide a unique means for exploring the gene–environment interactions that likely are involved in the genesis of PD and AD. Ideally such a study should enroll subjects at or even before conception and follow them through old age and should incorporate numerous biologic makers of exposure as well as detailed evaluations of behavioral and lifestyle factors, including information on occupational exposures and pesticide use. Such a prospective design would permit the real-time assessment of exposures as they occur and avoid the need for retrospective re-creation of past exposures. These features are now incorporated into the proposed National Children’s Study.

Four factors that make this a propitious time to launch a massive prospective epidemiologic study of the impact of the environment on health and development, such as the National Children’s Study, are *a*) the development of better skills in conducting and analyzing data from large prospective studies; *b*) the refinement of highly sensitive, extremely accurate chemical analyses that permit detection and quantification of xenobiotics in body fluids even at very low levels; *c*) advances in information technology; and *d*) capacity for rapid, relatively inexpensive genetic analysis (Berkowitz et al. 2001).

### Establish registries for Parkinson and Alzheimer patients.

Current data sources that rely principally on mortality statistics likely undercount the number of persons with neurodegenerative diseases. It is important to foster collaborations among agencies and to create new links across databases in different regions of the country to better track incidence rates of these disorders.

### Pursue suspected links between environmental exposures and neurobehavioral disorders in unique, high-risk populations.

Targeted studies of persons with unique patterns of disease such as the residents of Guam (Kurland and Mulder 1954) or persons with unusual environmental exposures such as those exposed to MPTP (Langston et al. 1999) demonstrate the value of undertaking clinical and epidemiologic pursuit of disease clusters.

### Improve toxicity test methods to better assess chronic neurodegeneration (Slotkin 2004).

Too few chemicals are tested for chronic neurotoxicity, and those that are examined are typically studied under test protocols in which the chemicals are administered during adolescence and the animals sacrificed and studied 12–24 months later. Functional assessment of neurologic function is often not included. This approach misses the opportunity to study possible late effects of early exposures. To overcome these limitations in design, conferees recommended that the duration of toxicity testing protocols should be extended to incorporate administration of chemicals in early life ideally *in utero* or even before conception, coupled with lifelong follow-up. Such expanded protocols may also incorporate functional neurobehavioral test batteries as well as neuropathologic examinations of relevant areas of the brain (Landrigan et al. 2003).
